# A Brief Review of Computer-Assisted Approaches to Rational Design of Peptide Vaccines

**DOI:** 10.3390/ijms17050666

**Published:** 2016-05-04

**Authors:** Ashesh Nandy, Subhash C. Basak

**Affiliations:** 1 Centre for Interdisciplinary Research and Education, Jodhpur Park, Kolkata 700068, India; 2 Natural Resources Research Institute and Department of Chemistry & Biochemistry, University of Minnesota Duluth, Duluth, MN 55811, USA; sbasak@nrri.umn.edu

**Keywords:** peptide vaccines, computer assisted vaccine development, epitopes, alignment-free analysis, web based approaches, sequence descriptors, rational peptide vaccine design, dengue, chikungunya, rotavirus, Zika virus

## Abstract

The growing incidences of new viral diseases and increasingly frequent viral epidemics have strained therapeutic and preventive measures; the high mutability of viral genes puts additional strains on developmental efforts. Given the high cost and time requirements for new drugs development, vaccines remain as a viable alternative, but there too traditional techniques of live-attenuated or inactivated vaccines have the danger of allergenic reactions and others. Peptide vaccines have, over the last several years, begun to be looked on as more appropriate alternatives, which are economically affordable, require less time for development and hold the promise of multi-valent dosages. The developments in bioinformatics, proteomics, immunogenomics, structural biology and other sciences have spurred the growth of vaccinomics where computer assisted approaches serve to identify suitable peptide targets for eventual development of vaccines. In this mini-review we give a brief overview of some of the recent trends in computer assisted vaccine development with emphasis on the primary selection procedures of probable peptide candidates for vaccine development.

## 1. Introduction

Viral diseases affect millions of people worldwide. Annually, dengue virus disease affects about 50 to 100 million people globally with 9000+ fatalities [1], rotavirus infects about two million children under five years of age, of whom about 527,000 die [2], seasonal influenza epidemics cause severe illness in three to five million people, and a quarter to a half million deaths [3], just to name a few. While drugs and vaccines are available for many of the viral diseases, the high mutation rate characteristic of viral genomes renders many of these rapidly obsolete. There is thus a continuous hunt for new drugs and vaccines, and this is compounded by the fact that new viruses are coming up to attack human hosts with higher frequency, while mutability of viral sequences rapidly render existing drugs and vaccines obsolete. Among the latest incidents of viral epidemics, one may recall the H1N1 (Influenza A type with Hemagglutinin subtype 1 and Neuraminidase subtype 1) swine flu pandemic of 2009, the SARS (Severe Acute Respiratory Syndrome) epidemic of 2002–2003 [4], the MERS (Middle East Respiratory Syndrome) epidemic of 2015 [5], the Ebola epidemic of 2014–2015 with 28,639 cases and 11,316 deaths reported until 16 March 2016 [6], the dengue epidemics in India of 2015, and now, in 2016, the Zika virus epidemic in South America.

## 2. Interest in Peptide Vaccines

The process of drug discovery from the bench to the market is long and expensive—more like 10 years and close to two billion dollars [7]. Since the viral epidemics die out within one year or two, such a process would be impossible for new viruses. Drugs are curatives; vaccines, as preventive means, have been a more attractive alternative, and development costs are relatively lower [8], but the standard practice of utilizing inactivated or attenuated viruses for the purpose has been fraught with their own problems including allergenic reactions [9].

Traditional vaccines, such as attenuated vaccines, also known as “live-attenuated” vaccines, are created by altering the genome, such that they become less virulent to harmless; vaccines against measles, mumps, rubella and others are created this way and have had a good success rate, but they have sometimes reverted to a virulence status through mutation [10]. Another conventional vaccine is the inactivated vaccine, which is produced by killing the original virus through heat or chemicals and then introducing the remaining virus shell into the host body. The shell, the virion capsid, when properly manufactured, retains enough of the original capsid to elicit immune response. Some varieties of polio vaccines and influenza vaccines are produced in this way. Improper manufacturing can retain some of the original virus and cause infections, and there are other problems, such as need for booster doses, *etc.*, as well.

A third type of vaccine manufactured from the original virus is the viral-like particle (VLP) vaccine. VLPs are constructed out of surface proteins that can self-assemble to a virus-like structure, which mimics the original virus structure and can elicit strong immune response with adjuvants. The first VLP vaccine license was granted by the US Food and Drug Administration for the hepatitis B virus in 1986 [11]; since then, two more licenses have been granted—for the human papillomavirus, and hepatitis E virus.

To cater to fast development and safer products, a new paradigm is taking shape in vaccine development. The availability of genomic data, the advancements in bioinformatics, technology and computing resources and increased understanding of immune responses and immunogenetics are moving developments away from “one size fits all” product prescriptions to one where possibilities of orientation towards individual, community and population specificity can exist in new vaccine design [12]. This is deemed to be of increasing necessity in hyper-variable viruses like coronavirus, influenza, and the like, where traditional vaccines are failing to cope with the changes [12,13]. Rational design of vaccines and the science of “reverse vaccinology”, as a yet nascent culture, are pointers towards the future of vaccine development.

Peptide vaccines belong to one of these new categories. The idea is to scan the viral genome for the protein antigens that can elicit an immune response and then synthesize them into a peptide vaccine. A more focused approach is to precisely locate the epitope regions within these antigens and utilize them to elicit the immune response [14]. The recent advancements in the technological and bioinformatics fields enable computer-based approaches for this purpose. Peptide vaccines have been in use against animals for some time. The first reported success was with the virulent canine parovirus [15], stimulating development of other peptide vaccines against diseases, such as malaria [16] and classical swine fever virus [17] in animals, and vaccines for humans are in various stages of trials [18]. Recombinant DNA technology and naked DNA have been used to induce immune response against virus infections; synthetic peptide vaccines have been the other approach in peptide vaccinology [19]. These have prompted adaptations along similar lines to develop vaccines for new viruses; the NIAID (National Institute of Allergy and Infectious Diseases), under the NIH (National Institute of Health), USA, has taken up the case of the Zika virus with urgency and is pursuing several paths, including a DNA-based vaccine using a strategy similar to that of a flavivirus vaccine for West Nile Virus, a live-attenuated version of the Zika virus and a genetically engineered version of vesicular stomatitis virus, all of them are presently on the lab bench [20].

**Figure 1:**
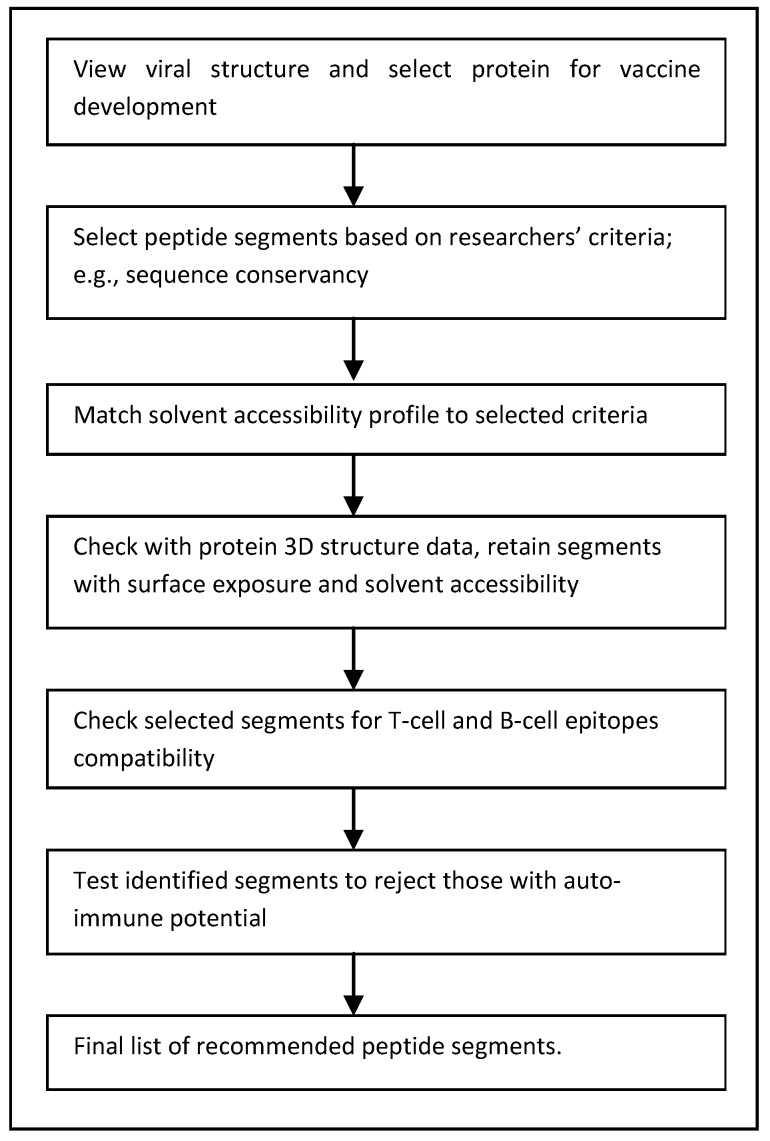
Work flow chart of peptide selection process, as in References [26,27,28].

The process of peptide vaccine determination involves identification of the appropriate viral protein and its peptide segments according to chosen criteria, ensuring adequate hydrophilicity of the selected peptides, epitope potential of the peptide segments against cellular and humoral immune response and tests to eliminate autoimmune threats. Then comes consideration of suitable carriers, questions of shelf life, and other logistical measures. Occasionally, multiple peptides for one or more viral infections can be combined into clusters, the multiple antigen peptide (MAP) for immunization. Such a MAP can be highly immunogenic and can substitute as a multivalent vaccine combining several selected peptides. Figure 1 gives a concise flow chart of peptide vaccine design.

The hypothesis of peptide vaccines has spawned many experiments for *in vivo* validation, sometimes straight from bioinformatics studies to the wet lab. Brossart *et al.* [21] showed that patients with advanced breast and ovarian cancers could benefit from MUC-1-derived peptide vaccines; MUC-1 is the gene in humans encoding cell surface associated mucin. Ludewig *et al.* [22] found protective antiviral and anti-tumor immune responses when a peptide antigen based vaccine against the lymphocytic choriomeningitis virus was administered intradermally. Liao *et al.* [23] of the Huazhong University of Science and Technology predicted the epitopes of human papillomavirus protein E5 as peptide vaccine candidates using bioinformatics study, and verified that, after administering the peptide along with a CpG (short single-stranded synthetic DNA molecule—cytosine triphosphate deoxynucleotide (“C”), a phosphodiester link ("p"), followed by a guanine triphosphate deoxynucleotide (“G”)) adjuvant by injection into muscles in a mouse model, strong cell-mediated immunity (CMI) and protection of the mice from tumor growth were seen. Likewise, Rojas-Caraballo *et al.* [24] determined several B-cell and T-cell epitope regions in Fasciola hepatica virus protein amino acid sequences using bioinformatics analyses; immunization of BALB/c mice (an albino, laboratory-bred strain of the House Mouse widely used in animal experiments) with synthetic peptides showed a high level of protection against the disease. A phase I trial of a multivalent peptide vaccine against non-small cell lung cancer found the vaccine using a mixture of four peptides to be safe and capable of generating strong T-cell responses [25].

## 3. Computational Approaches to Peptide Vaccines

The recent advances in bioinformatics, proteomics, immunoinformatics, structural biology and others have led to vaccinomics [29] and reverse vaccinology [30,31] as novel approaches for a generation of new vaccines. In the realm of drug development, scientific and technological advances have led to powerful inhibitors like Relenza for influenza being developed from the neuraminidase crystal structure [32] and AIDS (acquired immune deficiency syndrome) drugs, such as Aegenerase and Viracept, being developed from a structure-based design approach [33]. Advances in peptide-based vaccines have come from a better understanding of immunogenetics, antigenic MHC (Major Histocompatibility Complex) binding peptides, HLA (Human Leukocyte Antigen) binding motifs, and others. The NIH website of database of clinical studies of human participants [18] lists 559 peptide vaccines under various phases of trial and development, the vast majority of them (438) related to cancers. They include a recombinant protein comprising nine conserved peptides from influenza A and B as anti-influenza peptide vaccine in a phase IIb study [34], a phase I study of another influenza vaccine [35], a multi-peptide mix with adjuvant of newly discovered conserved segments from a HIV-1 protein to augment the body’s natural immunity with a broader, more rationally-designed immunity inducer [36], a phase 1 study by the Mayo Clinic [37] of combining a vaccine therapy with chemotherapy (cyclophosphamide) expecting higher efficiency in killing tumor cells, an ongoing phase 1 study by the US National Cancer Institute of peptide vaccines in treating patients with metastatic cancer who have not responded to previous therapy [38], among others. Singluff [39], in fact, had reported that peptide vaccines have shown good promise in the case of many cancers, and a mixture of peptides evokes a good immune response in 100% of the patients. Table 1 lists a few more peptide vaccine projects in various phases of clinical trials [18]. The large number of trials being undertaken by so many commercial firms and academic- and health-related institutions suggests the potential of, and interest in, peptide vaccines.

The rising interest in peptide vaccines has drawn many reviews [9,19,40,41,42,43,44], which may be referred to for the various aspects of this promising field. We are interested here in reviewing different approaches to one important step in the process of designing peptide vaccines, *viz*., choice of the protein and peptide segments. This is a crucial first step in the process, and good reliable results, firstly, require adequate molecular level data for each virus, and, second, a reliable technique to analyze the data to identify sequence segments of interest for the vaccine development purpose. Computer-based methodologies are the primary *modus operandi* here, as we had remarked in a recent article [45]. In this review, we elaborate on the bioinformatics and analytical approaches that have been used to pinpoint the peptide segments of choice.

### 3.1. Web-Server-Based Peptide Identification

With the growth of web accessibility and expanding entries in biological databases, numerous servers have been developed for various purposes. *In silico* screening of genomic information has reduced the workload of experimentalists with more focused goals. A number of server-based tools are available for prediction of probable antigenic sites, and their success is dependent on the accuracy of their predictions. While most approaches use sequence alignment to identify antigens, this approach has its limitations since some proteins may have additions, deletions and/or substitutions, but similar functions within the overall structure of the sequence, and these methods obviously cannot predict new and hitherto unrecognized antigens. This has given rise to alignment-free approaches, as in VaxiJen [46], which is based on auto cross-covariance, allowing for antigen classification solely based on the physicochemical properties of proteins collected from bacterial, viral, and tumor protein datasets.

Several web servers have been designed for predicting T-cell and B-cell epitopes targeting humoral- and cell-based immune responses. Popular web servers include IEDB (Immune Epitope Database) Analysis Resource [47], a curated database of experimentally characterized immune epitopes to compare the query peptides, and ABCpred (Artificial neural network based B-cell epitope prediction) server [48], which uses an artificial neural network program trained on a set of 700 known B-cell epitopes and 700 non-B-cell (*i.e.*, random) peptides to analyze the query peptide and predict its likelihood to be a B-cell epitope. PREDIVAC (prediction software for vaccine design) [49] was developed recently to predict CD4^+^ T-cell epitopes and was tested for high-affinity HLA class II peptide binding. It was found to compare well with several other web-accessible methods for HLA class II peptide-binding prediction, such as MHC2PRED (support vector machine based method for prediction of promiscuous MHC class II binding peptides).

Oany *et al.* [50] investigated a computational approach for the design of peptide vaccines against human coronavirus (HCoV), which causes upper respiratory tract infections and led to the SARS epidemic early this century. They presented 56 strains of the HCoV spike protein to the VaxiJen 2.0 server and selected the one with the highest antigenicity index for the next analysis of epitope-prediction for T-cell response. Selecting five peptides with the highest epitope scores from the protein, based on the results from the NetCTL 1.2 server [51], which predicts CTL (Cytotoxic T Lymphocytes) epitopes in protein sequences, they identified a 9 mer epitope, KSSTGFVYF amino acid sequence (a nonapeptide chain containing the following sequence of nine amino acids from left to right: lysine, serine, serine, threonine, glycine, phenylalanine, valine, tyrosine, phenylalanine), to interact with most MHC-I alleles with high affinity. They next determined the conservancy of the B-cell epitopes from the IEDB (Immune Epitope Database) server [26] and allergenicity from the AllerHunter tool and found this epitope to have 64.29% conservancy and an allergencity score well below threshold value. The next step was a molecular docking analysis, of the selected peptide with HLA-B*15:01, which was found to show good binding. A B-cell epitope search using the Kolaskar and Tongaonkar [27] antigenicity prediction method showed seven regions with high antigenic scores, which was reduced to three after solvent accessibility determination through the Emini surface accessibility option from the IEDB Analysis Resource. After further analysis with the Bprep epitope prediction server for linear B-cell epitopes, the authors concluded that the peptide GPSSQPY (a heptapeptide containg the amino acids from left to right: glycine, proline, serine, serine, glutamine, proline, tyrosine) was capable of inducing the desired immune response using B-cell epitopes.

A similar strategy was used by Islam *et al.* [28] to search for conserved high-scoring epitope regions in the proteins of the chikungunya virus. They did this by sequence alignment of selected strains of the virus, and then determining relative immune response propensities using different web servers. This enabled them to identify a stretch of conserved region in glycoprotein E2, which showed heightened T-cell and B-cell immunity potentials. Molecular docking studies further showed good binding of the epitope to the HLA.

Chakraborty *et al.* [52] approached peptide identification for dengue virus in a slightly different way. They determined the consensus sequence of the dengue virus envelope glycoprotein and nine conserved segments of 8 to 22 amino acids in each segment by sequence alignment of the complete envelope protein sequences of all four serotypes of the dengue virus and retaining only those segments where conservancy of the amino acids within each segment exceeded 50%. The next step was to discard those that had a hydrophobicity exceeding 50%, implying reduced propensity to surface exposure and, therefore, to antigen–antibody interaction, and ensure that those retained had high antigenicity, as determined through a web server like VaxiJen; the highest antigenicity (1.4911, as per VaxiJen) and low hydrophobicity (33.33%) was scored by the peptide FAGHLKCRL (amino acid sequence reading from left to right: phenylalanine, alanine, glycine, histidine, leucine, lysine, cysteine, arginine, leucine) out of the six total peptides recommended.

### 3.2. Software-Based Peptide Identification

Peptide vaccines against plant viruses, such as the alfalfa mosaic virus and cabbage leaf curl virus, have been identified with the help of neural networks. In Gomase *et al.* [53,54] neural networks trained on C terminals of known epitopes are used to predict MHC class I and II binding on viral protein peptide segments with high antigenic epitope properties. With such predicted binding properties to generate strong immune response, these antigens can be implemented in designing synthetic peptide vaccines.

### 3.3. Sequence-Descriptors-Based Peptide Identification

Mathematical approaches for the characterization of biomolecular sequences, a comparatively new area of sequence analysis research, have enabled alignment-free tools for rapid scanning of large numbers of sequences at a time to determine areas of similarities and dissimilarities. This has proven to be especially useful in virus-related studies, where the numerical measures have aided various studies, such as virus transmission paths [55], prognosis of possible reassortments in H5N2 bird flu [50,56], and phylogenetic analyses [57]. Ghosh *et al.* [58,59] used such alignment-free sequence descriptors to identify conserved peptides in viral protein sequences. The method is to scan each protein sequence by a window of 8/12/14 amino acids as required and consider the sequence descriptors in each window at each position for all the protein sequences and determine the regions with least variability. These regions, therefore, imply evolutionary pressures to retain their structure and, consequently, are relatively mostly conserved. These conserved stretches are analyzed for their hydrophilicity to determine the surface situated peptides, which are then confirmed through the protein 3D structures. The next step is to determine which ones have T-cell and B-cell epitopes, following which the retained peptides are tested for auto-immune threats. The remaining peptides that pass all these tests are reported to be good candidates to act as peptide vaccines.

Sarkar *et al.* [60], in their analysis on the hemagglutinin of the human-infecting H7N9 influenza, in 2013, in China, determined several target regions for the design of peptide vaccines against the H7N9 virus, and also were able to show through molecular docking and other analyses, that two mutations in the conserved region at the receptor binding site are characteristic of the human-infecting nature of H7N9.

## 4. Improving the Search for Peptide Vaccines

### 4.1. Computational Efficiency

An important consideration for computer-assisted drug and vaccine design is the power of the computer, as measured by its storage capacity, memory, and processing speed. In a recent exercise to calculate sequence descriptors for the genomic sequence of the Zika virus, which is 10,700 bases long in a matrix-oriented numerical characterization model, the time taken on a laptop computer with 4 GB RAM (4 gigabyte random access memory) and a dual core processor was still too long and had to be abandoned. The computational model, the processing power of the machine, and program efficiency are all factors to be considered when taking this approach to determine peptide vaccine targets.

In this context, it may be worthwhile to consider whether the techniques of Quantitative Structure-Activity Relationship (QSAR) can be extended to the search for suitable peptides. Mathematical biodescriptors derived from toxicoproteomics maps, and chemodescriptors used to predict their toxicity [61], could conceivably be extended to cover epitope binding. Integrated QSARs [62], developed using chemodescriptors for ligands and biodescriptors covering transcriptional, translational, and post-translational modification processes, connect structural information of DNA/RNA sequences, RNA secondary structures, and protein tertiary structures, and may be used to predict parameters for new entities [63]. It has been found that using numerical indices derived from protein—2-dimensional molecular graphics for QSAR studies is simpler than having to work with the 3D protein structures [64,65], and could be extended to optimize the search for surface antigens.

### 4.2. Bioinformatics Data

A primary requirement for the analysis of viral proteins to select segments suitable as vaccine targets is adequate data of the molecular sequences. The data needed are, for the purposes of this review, nucleotide sequence data, protein sequence data, and 3D structures of the associated proteins; other data useful for furthering analysis would comprise MHC binding data, epitope data, *etc*.

In the case of many viruses, there are enough data in the repositories; e.g., data are available, in abundance, for the main surface proteins of influenza A viruses and for rotavirus sequence data for surface proteins. There is, understandably, less 3D structural data, but, for these viruses, there is still adequate and varied data for the purpose of vaccine design. However, this is not so for many other viruses: It is understandable that adequate data are not available for the recently-emerged Zika virus, or the Ebola virus for that matter when it surfaced in a pathogenic epidemic form in 2015. However, in some cases, e.g., papillomavirus, the data are available in specialized databases where the data could be made available more widely if at least the primary data were to be represented in general databases like GenBank. Wider dissemination could lead to more research and perhaps new insights.

The other point to note is the quality of data. Maintaining high quality and data integrity has to be a prime requisite for numerical analyses, because erroneous data can throw computations out of gear and cause a loss of precious time when dealing with infections of epidemic proportions. In the case of the Zika genomic data, we noticed a rather large number of ambiguous or unconfirmed bases in the nucleotide sequences, which can cause erroneous results in some sensitive cases.

### 4.3. Transition to Wet Lab

While this review is about the computational aspects of the design of a new generation of vaccines, it is important to understand that translation of the bioinformatically-identified peptide to a working vaccine involves many steps. One of the major steps is identification of adjuvants. The presence of proper adjuvants greatly enhances immune response and adjuvnts are to be found integrated with peptide vaccines, and also VLP vaccines, such as Cerverix, which uses AS04 as an adjuvant. Almeida *et al.* [66] reported a strong immune response when using gold nano-particles as adjuvants in an ovalbumin peptide antigen leading to anti-tumor activity. Another problem to take note of is the possibility of the peptides folding up and, therefore, sharply reducing immune activity. Storage and transport are other factors that need to be investigated before a peptide vaccine can be made ready for the market, but these deliberations are far outside the scope of this review.

## 5. Conclusions

Computer-assisted drug and vaccine discovery is a relatively new field, where many aspects are still in progress. The development of many web-assisted platforms for analysis of various aspects of the process of selecting the final determinant of an immune response generator against causative agents has automated several tasks. However, the methods for initial screening of probable vaccine candidates are a matter of choice and pure *in silico* techniques, coupled with biodescriptors and other mathematical methods, are still relatively novel. Overall, wet-lab experimentations are necessary to prove the viability of this new approach. However, the relative advantages of peptide vaccine formulations in terms of speed and cost of development, and the frequent incidences of viral epidemics and new varieties of viral diseases, make the pursuit of a rational design of peptide vaccines an important alternative. In the endeavor, computer-assisted approaches appear to show good promise for peptide vaccine identification strategies.

## Figures and Tables

**Table 1:** A selection of peptide vaccines in various trial stages from the NIH website [18].

Peptide Vaccine/Disease	Firm/Institution	Trial Stage	Reference
BOPRIVA (^®^ Registered Trade Mark name) peptide vaccine induces antibodies against Gonadotropin-releasing factor and controls aggressive behavior in bulls	Pfizer	launched	[9]
NeuVax synthetic peptide vaccine for breast cancer prevention	Galena Biopharma,	Phase III	Identifier: NCT01479244 in [18]
Peptide vaccine for HIV (human immunodeficiency virus)	United Biomedical	Phase I/II completed	Identifier: NCT00002428 in [18]
Peptide vaccine for recurrent prostate cancer	University Hospital Tuebingen	Phase II	Identifier: NCT02452307 in [18]
Multileric linear epitope based peptide vaccine for influenza A and B	BiondVax Pharmaceuticals Ltd.	Phase II	Identifier: NCT02691130 in [18]

## Abbreviations

DOAJ	Directory of open access journals
MHC	Major Histocompatibility Index
SARS	Severe Acute Respiratory Syndrome
MERS	Middle East Respiratory Syndrome
NIAID	National Institute of Allergy and Infectious Diseases
NIH	National Institute of Health
MAP	Multiple Antigen Peptide
AIDS	Auto Immunity Deficiency Syndrome
HLA	Human Leukocyte Antigen
IEDB	Immune Epitope Database
ABCpred	Artificial neural network based B-cell epitope prediction
HCoV	Human Coronavirus
CTL	Cytotoxic T Lymphocytes
GB	Gigabytes
RAM	Random Access Memory
QSAR	Quantitative Structure Activity Relationship

## References

[1] Stanaway J.D., Shepard D.S., Undurraga E.A., Halasa Y.A., Coffeng L.E., Brady O.J., Hay S.I., Bedi N., Bensenor I.M., Castañeda-Orjuela C.A. (2016). The global burden of dengue: An analysis from the Global Burden of Disease Study 2013. Lancet Infect. Dis..

[2] World Health Organization (2008). Global networks for surveillance of rotavirus gastroenteritis, 2001–2008. Wkly. Epidemiol. Rec..

[3] World Health Organization (2014). Influenza (Seasonal). http://www.who.int/mediacentre/factsheets/fs211/en/.

[4] Gorbalenya A.E., Snijder E.J., Spaan W.J. (2004). Severe acute respiratory syndrome coronavirus phylogeny: Toward consensus. J. Virol..

[5] World Health Organization (2015). Middle East Respiratory Syndrome Coronavirus (MERS-CoV). http://www.who.int/mediacentre/factsheets/mers-cov/en/.

[6] World Health Organization (2016). Ebola Virus Disease Outbreak. http://www.who.int/csr/disease/ebola/en/.

[7] PR Tufts CSDD 2014 Cost Study Cost to Develop and Win Marketing Approval for a New Drug Is $2.6 Billion. http://csdd.tufts.edu/news/complete_story/pr_tufts_csdd_2014_cost_study.

[8] Chit A., Parker J., Halperin S.A., Papadimitropoulos M., Krahn M., Grootendorst P. (2014). Toward more specific and transparent research and development costs: The case of seasonal influenza vaccines. Vaccine.

[9] Wong S.-S., Webby R.J. (2013). Traditional and new influenza vaccines. Clin. Microbiol. Rev..

[10] Shimizu H., Thorley B., Paladin F.J., Brussen K.A., Stambos V., Yuen L., Utama A., Tano Y., Arita M., Yoshida H. (2004). Circulation of type 1 vaccine-derived poliovirus in the Philippines in 2001. J. Virol..

[11] Chroboczek J., Szurgot I., Szolajska E. (2014). Virus-like particles as vaccine. Acta Biochim. Polonica.

[12] Poland G.A., Whitaker J.A., Poland C.M., Ovsyannikova I.G., Kennedy R.B. (2016). Vaccinology in the third millennium: Scientific and social challenges. Curr. Opin. Virol..

[13] Poland G.A., Kennedy R.B., Ovsyannikova I.G. (2011). Vaccinomics and personalized vaccinology: Is science leading us toward a new path of directed vaccine development and discovery?. PLoS Pathog..

[14] Purcell A.W., McCluskey J., Rossjohn J. (2007). More than one reason to rethink the use of peptides in vaccine design. Nat. Rev..

[15] Langeveld J.P.M., Casal J.I., Osterhaus A.D.M.E., Cortes E., de Swart R., Vela C., Dalsgaard K., Puijk W.C., Schaaper W.M.M., Meloen R.H. (1994). First peptide vaccine providing protection against viral infection in the target animal: Studies of canine parvovirus in dogs. J. Virol..

[16] Wang R., Charoenvit Y., Corradin G., Porrozzi R., Hunter R.L., Glenn G., Alving C.R., Church P., Hoffman S.L. (1995). Induction of protective polyclonal antibodies by immunization with a Plasmodium yoelii circumsporozoite protein multiple antigen peptide vaccine. J. Immunol..

[17] Monso M., Tarradas J., de la Torre B.G., Sobrino F., Ganges L., Andreu D. (2011). Peptide vaccine candidates against classical swine fever virus: T cell and neutralizing antibody responses of dendrimers displaying E2 and NS2–3 epitopes. J. Pept. Sci..

[18] ClinicalTrials gov https://clinicaltrials.gov/ct2/results?term=peptide+vaccines&amp;pg=2.

[19] Moisa A.A., Kolesanova E.F., Roy P. (2012). Synthetic peptide vaccines. Insight and Control of Infectious Disease in Global Scenario.

[20] NIAID https://www.niaid.nih.gov/topics/zika/Pages/default.aspx.

[21] Brossart P., Wirths S., Stuhler G., Reichardt V.L., Kanz L., Brugger W. (2000). Induction of cytotoxic T-lymphocyte responses *in vivo* after vaccinations with peptide-pulsed dendritic cells. Blood.

[22] Ludewig B., Barchiesi F., Pericin M., Zinkernagel R.M., Hengartner H., Schwendener R.A. (2001). *In vivo* antigen loading and activation of dendritic cells via a liposomal peptide vaccine mediates protective antiviral and anti-tumour immunity. Vaccine.

[23] Liao S.-J., Deng D.-R., Zeng D., Ma D. (2013). HPV16 E5 peptide vaccine in treatment of cervical cancer *in vitro* and *in vivo*. J. Huazhong Univ. Sci. Technol..

[24] Rojas-Caraballo J., López-Abán J., Pérez del Villar L., Vizcaíno C., Vicente B., Fernández-Soto P., del Olmo E., Patarroyo M.A., Muro A. (2014). *In vitro* and *in vivo* studies for assessing the immune response and protection-inducing ability conferred by fasciola hepatica-derived synthetic peptides containing B- and T-cell epitopes. PLoS ONE.

[25] Suzuki H., Fukuhara M., Yamaura T., Mutoh S., Okabe N., Yaginuma H., Hasegawa T., Yonechi A., Osugi J., Hoshino M. (2013). Multiple therapeutic peptide vaccines consisting of combined novel cancer testis antigens and anti-angiogenic peptides for patients with non-small cell lung cancer. J. Trans. Med..

[26] IEDB Analysis Resource. http://tools.immuneepitope.org/bcell/.

[27] Kolaskar A.S., Tongaonkar P.C. (1990). A semi-empirical method for prediction of antigenic determinants on protein antigens. FEBS Lett..

[28] Islam R., Sakib M.S., Zaman A. (2012). A computational assay to design an epitope-based peptide vaccine against chikungunya virus. Future Virol..

[29] Poland G.A., Ovsyannikova I.G., Jacobson R.M. (2009). Application of pharmacogenomics to vaccines. Pharmacogenomics.

[30] Rappuoli R. (2001). Reverse vaccinology, a genome-based approach to vaccine development. Vaccine.

[31] Barocchi M.A., Censini S., Rappuoli R. (2007). Vaccines in the era of genomics: The pneumococcal challenge. Vaccine.

[32] Varghese J.N. (1999). Development of neuraminidase inhibitors as anti-influenza virus drugs. Drug Dev. Res..

[33] Hardy L.W., Malikayil A. (2003). The impact of structure-guided drug design on clinical agents. Curr. Drug Discov..

[34] Assess the Safety and Immunogenicity of M-001 as A Standalone Influenza Vaccine and as A H5N1 Vaccine Primer in Adults. https://clinicaltrials.gov/ct2/show/NCT02691130?term=influenza+AND+peptide+vaccines&amp;rank=4.

[35] Safety, Tolerability and Immunogenicity of Two Different Formulations of an Influenza A Vaccine (FP-01.1). https://clinicaltrials.gov/ct2/show/NCT01677676?term=influenza+AND+peptide+vaccines&amp;rank=9.

[36] HIV-1 Peptide Immunisation of Individuals in West Africa to Prevent Disease (HIV-BIS). https://clinicaltrials.gov/ct2/show/NCT01141205?term=sers+peptide+vaccine&amp;rank=12.

[37] Vaccine Therapy and Cyclophosphamide in Treating Patients with Stage II-III Breast or Stage II-IV Ovarian, Primary Peritoneal, or Fallopian Tube Cancer. https://clinicaltrials.gov/ct2/show/NCT01606241?term=cancer+and+peptide+vaccines&amp;rank=4.

[38] Vaccine Therapy in Treating Patients with Metastatic Cancer. https://clinicaltrials.gov/ct2/show/NCT00020267?term=cancer+and+peptide+vaccines&amp;rank=16.

[39] Singluff C.L. (2011). The present and future of peptide vaccines for cancer: Single or multiple, long or short, alone or in combination?. Cancer J..

[40] Huber S.R., van Beek J., de Jonge J., Luytjes W., van Baarle D. (2014). T cell responses to viral infections—Opportunities for peptide vaccination. Front. Immunol..

[41] Li W., Joshi M.D., Singhania S., Ramsey K.H., Murthy A.K. (2014). Peptide vaccine: Progress and challenges. Vaccines.

[42] Sirskyj D., Diaz-Mitoma F., Golshani A., Kumar A., Azizi A. (2011). Innovative bioinformatic approaches for developing peptide-based vaccines against hypervariable viruses. Immunol. Cell Biol..

[43] Sobolev B.N., Olenina L.V., Kolesanova1 E.F., Poroikov V.V., Archakov A.I. (2005). Computer design of vaccines: Approaches, software tools and informational resources. Curr. Comp. Aided Drug Des..

[44] Tomar N., de R.K. (2010). Immunoinformatics: An integrated study. Immunology.

[45] Basak S.C., Nandy A. (2016). Computer-assisted approaches as decision support systems in the overall strategy of combating emerging diseases: Some comments regarding drug design, vaccinomics, and genomic surveillance of the Zika virus. Curr. Comput.-Aided Drug Des..

[46] Doytchinova I.A., Flower D.R. (2007). VaxiJen: A server for prediction of protective antigens, tumour antigens and subunit vaccines. BMC Bioinform..

[47] Immune Epitope Database and Analysis Resource. http://www.iedb.org/home_v2.php?Clear=Clear#.

[48] ABCpred. http://www.imtech.res.in/raghava/abcpred/.

[49] Oyarzun P., Ellis J.J., Bodén M., Kobe B. (2013). PREDIVAC: CD4^+^ T-cell epitope prediction for vaccine design that covers 95% of HLA class II DR protein diversity. BMC Bioinform..

[50] Oany A.R., Emran A.-A., Jyoti T.P. (2014). Design of an epitope-based peptide vaccine against spike protein of human coronavirus: An *in silico* approach. Drug Des. Dev. Ther..

[51] NetCTL 1.2 Server. http://www.cbs.dtu.dk/services/NetCTL/.

[52] Chakraborty S., Chakravorty R., Ahmed M., Rahman A., Waise T.M.Z., Hassan F., Rahman M., Shamsuzzaman S. (2010). A computational approach for identification of epitopes in dengue virus envelope protein: A step towards designing a universal dengue vaccine targeting endemic regions. In Silico Biol..

[53] Gomase V.S., Kale K.V., Chikhale N.J., Changbhale S.S. (2007). Prediction of MHC binding peptides and epitopes from alfalfa mosaic virus. Curr. Drug Discov. Technol..

[54] Gomase V.S., Kale K.V. (2008). Prediction of MHC binder for fragment based viral peptide vaccines from cabbage leaf curl virus. Gene Ther. Mol. Biol..

[55] Ghosh A., Nandy A., Nandy P., Gute B.D., Basak S.C. (2009). Computational study of dispersion and extent of mutated and duplicated sequences of the H5N1 influenza neuraminidase over the period 1997–2008. J. Chem. Inf. Model..

[56] Nandy A., Basak S.C. (2015). Prognosis of possible reassortments in recent H5N2 epidemic influenza in USA: Implications for computer-assisted surveillance as well as drug/vaccine design. Curr. Comput. Aided Drug. Des..

[57] Liao B., Liu Y., Li R., Zhu W. (2006). Coronavirus phylogeny based on triplets of nucleic acids bases. Chem. Phys. Lett..

[58] Ghosh A., Nandy A., Nandy P. (2010). Computational analysis and determination of a highly conserved surface exposed segment in H5N1 avian flu and H1N1 swine flu neuraminidase. BMC Struct. Biol..

[59] Ghosh A., Chattopadhyay S., Chawla-Sarkar M., Nandy P., Nandy A. (2012). *In silico* study of rotavirus VP7 surface accessible conserved regions for antiviral drug/vaccine design. PLoS ONE.

[60] Sarkar T., Das S., De A., Nandy P., Chattopadhyay S., Chawla-Sarkar M., Nandy A. (2015). H7N9 influenza outbreak in China 2013: *In silico* analyses of conserved segments of the hemagglutinin as a basis for the selection of peptide vaccine targets. Comput. Biol. Chem..

[61] Hawkins D.M., Basak S.C., Kraker J., Geiss K.T., Witzmann F.A. (2006). Combining chemodescriptors and biodescriptors in quantitative structure-activity relationship modeling. J. Chem. Inf. Model..

[62] Basak S.C., Mills D., Gute B.D., Natarajan R., Gupta S.P. (2006). Predicting pharmacological and toxicological activity of heterocyclic compounds using QSAR and molecular modeling. QSAR and Molecular Modeling Studies of Heterocyclic Drugs, I.

[63] Gonzalez-Diaz H., Gonzalez-Diaz Y., Santana L., Ubeira F.M., Uriarte E. (2008). Proteomics, networks and connectivity indices. Proteomics.

[64] Gonzalez-Diaz H., Perez-Montoto L.G., Duardo-Sanchez A., Paniagua E., Vazquez-Prieto S., Vilas R., Dea-Ayuelac M.A., Bolas-Fernándezd F., Munteanue C.R., Doradoe J. (2009). Generalized lattice graphs for 2D-visualization of biological information. J. Theor. Biol..

[65] Vilar S., González-Díaz H., González-Díaz H., Munteanu C.R. (2010). QSPR models for human Rhinovirus surface networks. Topological Indices for Medicinal Chemistry, Biology, Parasitology, Neurological and Social Networks.

[66] Almeida J.P., Lin A.Y., Figueroa E.R., Foster A.E., Drezek R.A. (2015). *In vivo* gold nanoparticle delivery of peptide vaccine induces anti-tumor immune response in prophylactic and therapeutic tumor models. Small.

